# The Staff of Bermondsey Hospital

**Published:** 1920-03-13

**Authors:** Henry H. Reeve

**Affiliations:** Clerk to the Parish of Bermondsey. 283 Tooley Street, S.E. 1.


### THE EDITOR'S LETTER-BOX.

**(Correspondence on all subjects is invited, but we cannot in any way be responsible for the opinions expressed by our correspondents, who must give their name and address as a guarantee of good faith, but not necessarily for publication. Correspondents are reminded that brevity of style and conciseness of statement greatly facilitate early insertion.)**

### The Staff of Bermondsey Hospital.

*To the Editor of* THE HOSPITAL.

SIR,—My attention has been drawn to the statement contained in your issue of February 28, 1920, under the above heading that Dr. H. P. Ashe has been appointed first assistant medical officer at the Guardians' Infirmary at Rotherhithe. The gentleman appointed by the Guardians as first assistant medical officer at the infirmary was Dr. John Allen Munro Cameron, M.B., Ch.B. (Glasgow University), and not Dr. Ashe, as reported by you.—I am, Sir, your obedient servant, HENRY H. REEVE,

Clerk to the Parish of Bermondsey.

283 Tooley Street, S.E. 1.

[The information came to us from a trusted correspondent, and we regret he should have led us into error. Dr. Ashe was one of the three candidates selected to come before the Board, Drs. Cameron and Nolan being the other two, the former being appointed.—ED. T.H.]

